# Ablation of recurrent malignant idiopathic ventricular tachycardia: When proper diagnosis and success is a matter of contact

**DOI:** 10.1002/ccr3.1777

**Published:** 2018-10-05

**Authors:** Marzia Giaccardi, Giuseppe Mascia, Alessandro Paoletti Perini, Andrea Giomi, Stella Cartei, Andrea Colella

**Affiliations:** ^1^ Cardiology and Electrophysiology Unit Santa Maria Nuova Hospital Firenze Italy; ^2^ Electrophysiology Unit Clinica Montevergine Mercogliano Italy; ^3^ Electrophysiology Unit Azienda Ospedaliero Universitaria Careggi Firenze Italy

**Keywords:** ablation success, contact force catheter, radiofrequency ablation, ventricular tachycardia

## Abstract

Effective and stable contact between the catheter tip and the tissue is crucial for both mapping and lesion formation during cardiac ablation procedures. Contact force catheter may be not only a therapeutic approach to arrhythmias, but also a tool for achieving accurate characterization of the arrhythmic substrate.

## 1 INTRODUCTION

Despite significant improvements in catheter ablation strategies for the treatment of ventricular tachycardias (VTs), rate of ablation failure and recurrences remains high. A variety of factors have been reported to increase the risk of unfavorable postablation outcome, including incomplete procedural success, low left ventricular ejection fraction, VT storm, a high number of clinical VTs, a high number of failed antiarrhythmic drugs, and a long mean cycle length of induced VTs.^1^ The lack of contact force (CF) information might be another possible cause of procedural failure. Even in experienced operators’ hands, about 20% of ventricular endocardial radiofrequency (RF) applications do not result in adequate lesion formation, especially when fluoroscopy, tactile feedback, or electrogram amplitude are used to assess contact. The availability of CF information dramatically reduces the number of RF applications that fail to result in effective lesion formation.^2^ We report a case of idiopathic VT from the left ventricular septum for which formulation of the correct diagnosis, and subsequent effective ablation, was achieved only by means of an irrigated‐tip catheter that provided CF information.

## 2 CASE REPORT

**Figure 1:**
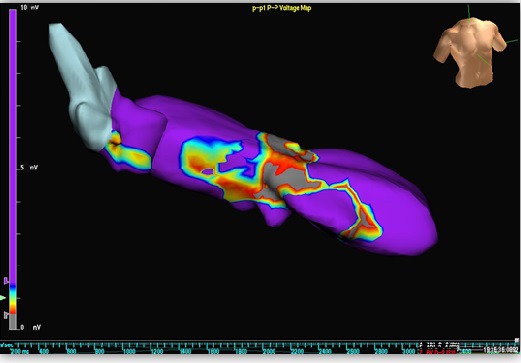
Scar region documented in the mid‐septum during the previous ablation procedure

**Figure 2:**
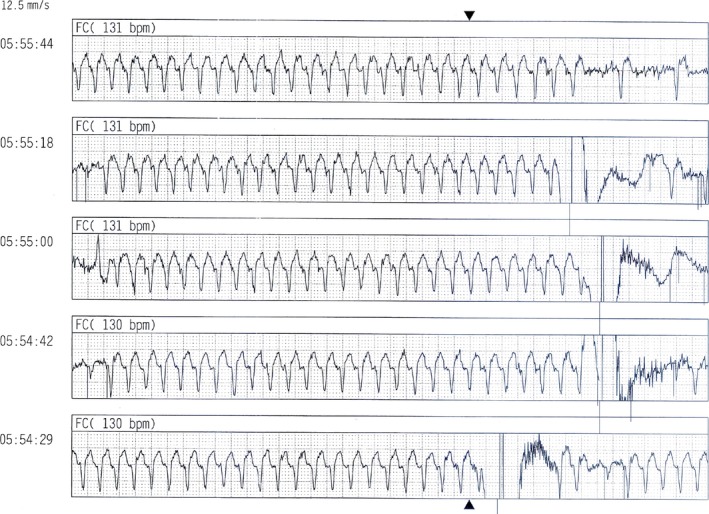
Several recurrences of ventricular tachycardia (VT) with arrhythmic storm

**Figure 3:**
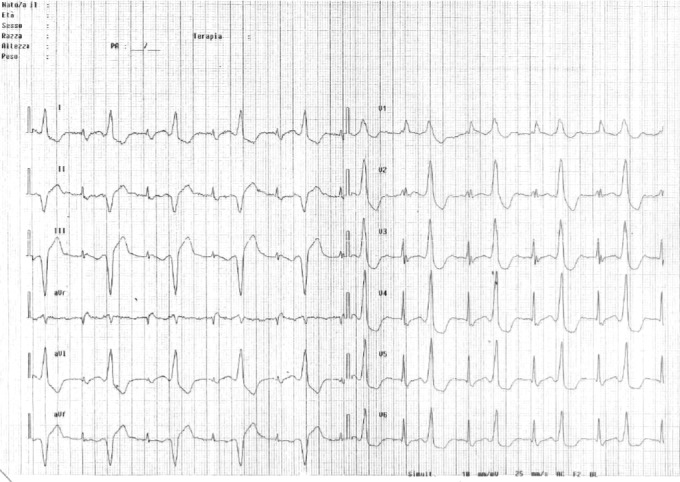
Premature ventricular contraction originating from the mid‐inferoseptum of the left ventricle

**Figure 4:**
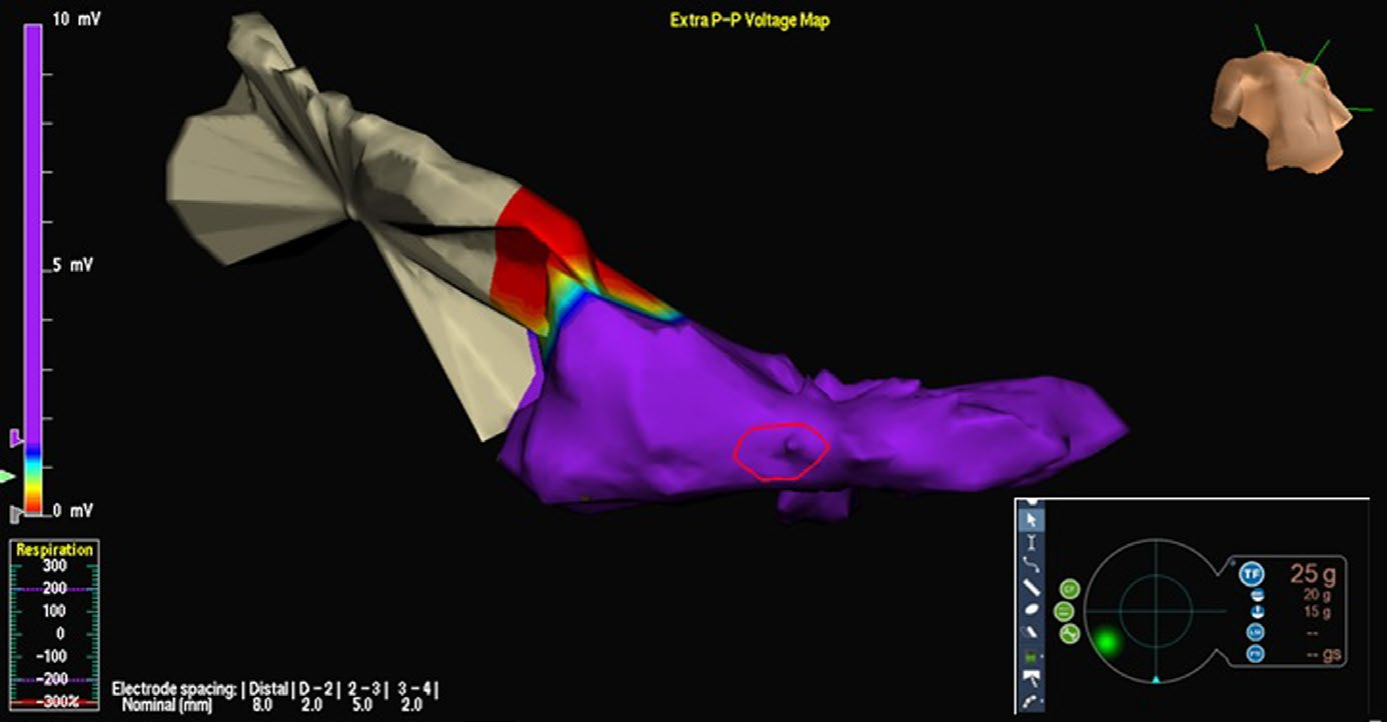
Voltage mapping during sinus rhythm, showing the presence of normal tissue with no identifiable lesion formation from previous radiofrequency applications. The voltage map was made with TactiCath; St. Jude Medical St. Paul, MN

**Figure 5:**
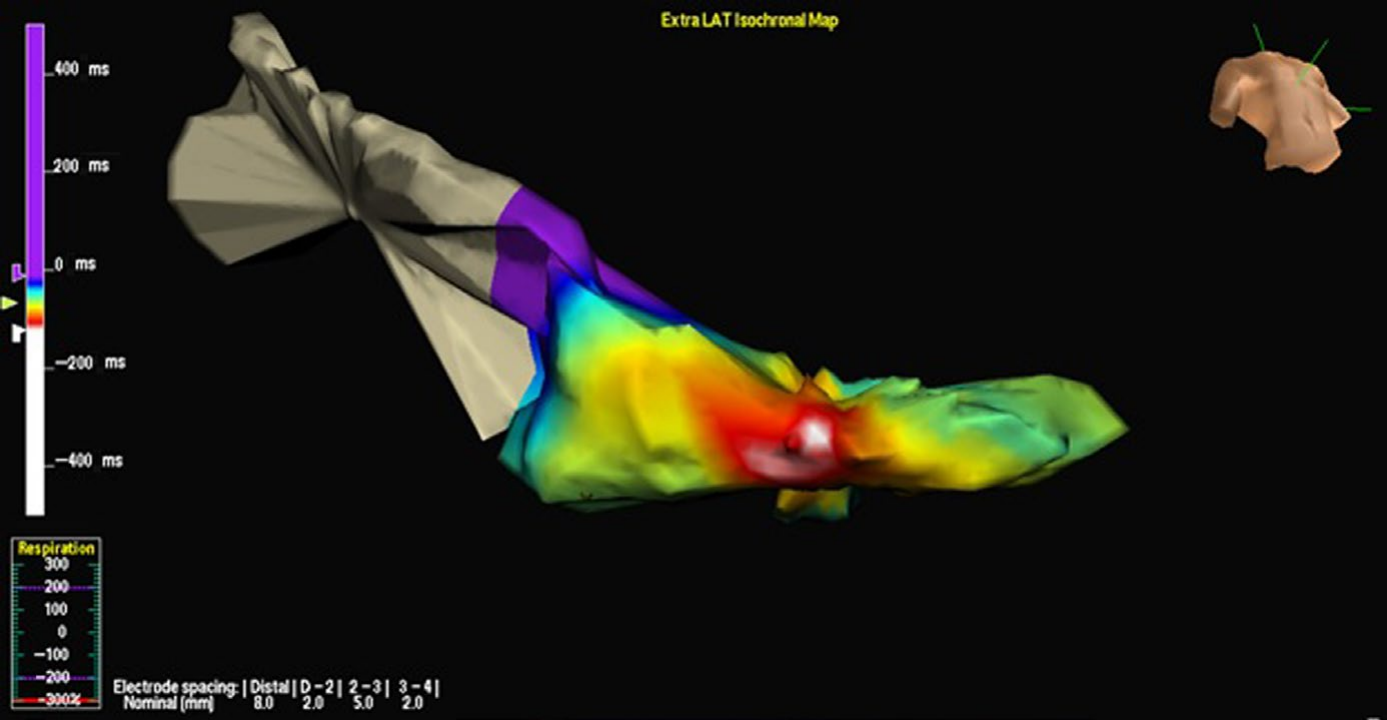
Activation mapping during the induced ventricular tachycardia (VT), showing a focal VT. Radiofrequency ablation was performed at the site of earliest activity

A 70‐year‐old man, previously implanted (in 2011) with a dual‐chamber implantable cardioverter‐defibrillator (ICD) in secondary prevention for symptomatic sustained idiopathic VT, no coronary artery disease, and preserved left ventricular (LV) ejection fraction, was referred to our hospital for electrophysiological study in January 2014. His history was notable for several ICD discharges due to incessant VTs since 2012, which were refractory to multiple antiarrhythmic agents. Therefore, the patient had undergone three previous ablation procedures in the same year (2012), the first at our center, the second and third in a different hospital. During all previous procedures, substrate bipolar voltage mapping of the left ventricle (Ensite Velocity System St Jude Medical, St. Paul, MN, USA) had been performed through a catheter without CF sensor, and the presence of a scar region (bipolar electrogram voltage ≤0.5 mV) had been documented in the mid‐inferoseptum (Figure 1). Ablations had been performed by means of the same mapping catheter, and with the same parameters (i.e., RF power 50 W with maximum temperature of 45°C and irrigation flow of 15 mL/min) using a retrograde transaortic approach. In all cases, the ablation strategy had been exclusively based on a substrate‐guided approach and pace‐mapping, due to the noninducibility of the clinical VT. During the following months, the patient had suffered several recurrences of VT, with three arrhythmic storms (Figure 2, cycle length: 460 ms), triggering ICD shocks (overall, up to 97 appropriate shocks since implantation). To the fourth hospital admission in January 2014, a 12‐lead ECG showed sinus rhythm and premature ventricular contraction originating from the mid‐inferoseptum of the left ventricle (Figure 3). At same time, transthoracic echocardiography revealed normal biventricular systolic function and mild‐to‐moderate mitral valve regurgitation. We decided to perform a new ablation procedure with a CF‐sensing catheter (TactiCath; St. Jude Medical, St. Paul, MN), via retrograde transaortic approach. Voltage mapping, performed during sinus rhythm, with an adequate electrode contact during mapping to avoid un underestimation of voltage (stable CF > 8 g)^3^ showed the presence of healthy tissue, no scar, and no signs of previous RF ablation lesions (Figure 4). A sustained monomorphic VT could be induced by means of programmed ventricular electrical stimulation (PES) at 450 ms of drive with three ventricular extrastimuli from the right ventricular apex under isoprenaline infusion. The VT morphology match to a clinical ventricular premature beats (Figure 3) in all 12 leads. Activation mapping during arrhythmia showed a focal VT (Figure 5). RF ablation was performed at the site of earliest activity, maintaining good and stable contact, with the same parameters of previous procedures (i.e., RF power 50 Watt with maximum temperature of 45°C and irrigation flow of 15 mL/min), CF between 10 and 20 g and min force‐time interval (FTI) >400 g. During a single RF ablation attempt, sinus rhythm was restored in 30 seconds; after RF delivery, the VT was no more inducible by PES. Procedure was not performed under anesthesia, and at same time, no complications were observed. After this last procedure, the patient remained free from any arrhythmic recurrences at the subsequent 48‐month follow‐up.

## 3 DISCUSSION

This case demonstrates the effectiveness of CF not only as a therapeutic approach to ventricular arrhythmias, but also as a tool for achieving accurate characterization of the arrhythmic substrate, especially when the endocardial areas involved are difficult to achieve. In our patient, the area of scar observed during the previous procedures did not reflect the real presence of an endocardial potential <0.5 mV, but it was rather expression of poor contact between the tissue and the catheter tip. This may lead to the misinterpretation of the arrhythmic substrate. In the first three procedures, the surrogate parameters, such as electrogram amplitude, impedance values, tactile feedback, and fluoroscopy, using for assessment of good contact, are inaccurate to identify the correct substrate.^4^ The difficulty of directly assessing the degree and quality of contact between the catheter tip and the target tissue has been demonstrated even in experienced operators.^2^ Moreover, preclinical research has shown that insufficient CF can result in ineffective lesion formation.^5^, ^6^, ^7^ Therefore, poorer clinical outcome can occur even if tactile feedback, fluoroscopy, and high‐amplitude electrograms appear to indicate good contact.^2^ In this regard, it can be hypothesized that failure to achieve adequate lesion formation after the previous RF applications in our patients was facilitated by the use of a catheter without CF. To the best of our knowledge, this is the first report of an idiopathic VT that was correctly diagnosed, and efficiently ablated, only by adding information from a CF catheter to the standard mapping procedure. In the present case, the VT was successfully eliminated and no tachycardia was documented during long‐term follow‐up (4 years), while after all previous attempts, recurrences had occurred thereafter, within few months, with a dramatic clinical presentation. This suggests that some cases of VT ablation failure might be due to the use of catheters without CF feedback, resulting in an unreliable map, incorrect diagnosis, and ineffective lesions.

## 4 CONCLUSIONS

During a VT ablation procedure, effective and stable contact between the catheter tip and the tissue is crucial for both mapping and lesion formation. Indirect strategies to assess contact may be unreliable and lead to incorrect diagnosis, or to ineffective ablation, which may have a deleterious impact on patients’ clinical outcome. Catheters able to provide CF information may help achieve either procedural target even after several previous ablation failures.

## CONFLICT OF INTEREST

None declared.

## AUTHORSHIP

MG, GM, APP, AG, SC, and AC: contributed equally to this case. MG, GM and AC: performed the procedure and equally contributed to write the manuscript. APP, AG, and SC: performed routinely patient's follow‐up at our institution.
